# Body Composition Evaluation in Head and Neck Cancer Patients: A Review

**DOI:** 10.3389/fonc.2019.01112

**Published:** 2019-11-07

**Authors:** Inês Almada-Correia, Pedro Miguel Neves, Antti Mäkitie, Paula Ravasco

**Affiliations:** ^1^Centre for Interdisciplinary Research in Health, Universidade Católica Portuguesa, Lisbon, Portugal; ^2^Department of Otorhinolaryngology-Head and Neck Surgery, Helsinki University Hospital and University of Helsinki, Helsinki, Finland; ^3^Research Programme in Systems Oncology, Faculty of Medicine, University of Helsinki, Helsinki, Finland; ^4^Division of Ear, Nose and Throat Diseases, Department of Clinical Sciences, Intervention and Technology, Karolinska Institutet and Karolinska University Hospital, Stockholm, Sweden; ^5^University Hospital of Santa Maria, Universidade de Lisboa, Lisbon, Portugal

**Keywords:** head and neck cancer, body composition, cachexia, lean body mass, BIA, DXA, CT

## Abstract

**Introduction:** Head and neck cancer (HNC) patients show a high risk of malnutrition due to the lifestyle habits adopted prior to the diagnosis as well as to the compromising impact of both the anatomical location of the tumor and the treatment modalities on food intake. Weight change, measurement of skinfold thickness, biochemical parameters, bioelectrical impedance analysis (BIA), computed tomography (CT), magnetic resonance (MRI), or dual-energy x-ray absorptiometry (DXA) are available techniques to evaluate nutritional status and/or body composition in the clinical practice. Evaluating body composition alterations in HNC patients is essential to be able to offer the best therapeutical interventions. In this paper, we review the existing literature regarding body composition evaluation in HNC patients to determine, which is the most suitable method for this population, regarding availability in the day-to-day practice, patient burden, cost, sensibility, and specificity.

**Methodology:** A literature search for relevant papers indexed in MEDLINE, Cochrane Library and Scielo was conducted, with no publication date restriction and for all published articles until the 31 January, 2019. All the papers written in English, with interventions in humans, exclusively considering HNC patients were selected.

**Results:** A total of 41 studies with different methodologies were included in this review. In 15 studies BIA was the used assessment method and three of them also evaluated skinfold thickness and one was a bioelectric impedance vector analysis (BIVA). Body composition assessment was made with DXA in eight studies, one of which also included muscle biopsies. In two studies the chosen method was both BIA and DXA. CT/ positron emission tomography-CT was applied in 11 studies and one also included MRI. In two studies body composition was assessed with skinfold measurements alone and one study only used BIVA.

**Conclusions:** Despite the different existing body composition assessment tools, it seems that skeletal muscle mass (SMM) measurement at the level of cervical spine C3 vertebra may be a reliable method for SMM assessment as it strongly correlates with cross-sectional area measures at the level of L3 and it allows a cost-effective body composition assessment without the need for additional radiation exposure.

## Introduction

Head and neck cancer (HNC) is responsible for ~300.000 annual deaths worldwide, with a 40–50% survival rate (1). Beside the hypercatabolic characteristics of cancer, HNC patients show a high risk of malnutrition due to the lifestyle habits, such as smoking and alcohol consumption, adopted prior to the diagnosis as well as to the compromising impact that both the anatomical location of the tumor and the various treatment modalities may have on food intake (2, 3). It is estimated that about 60% of these patients show poor nutritional status and about 80% lose weight during treatment (2, 3). Malnourished patients have a higher risk of infection, delayed wound healing, impaired cardiac and respiratory function, muscular weakness, depression, poor quality of life, higher rate of postoperative complications, higher risk of refeeding syndrome, impaired response to treatments, higher mortality rate as well as longer hospitalization time (2). In order to counter malnutrition, its' early detection is critical.

Some authors suggest that ~70% of the weight loss identified in HNC patients corresponds to lean body mass (LBM) (4–6). Loss of LBM has been presented as an important prognostic factor (4, 7) with influence in treatment toxicities (8), risk of recurrence and mortality (9), impaired muscle strength, a decline in physical activity and functional performance (6). It seems that, in HNC patients, LBM depletion may provide additional and relevant information as an outcome predictor beside weight loss alone and furthermore, high BMI, and low LBM may associate with each other (10). For these reasons, considering LBM as a predictor of clinical outcome would take into account also patients with sarcopenic obesity (11). The main component of LBM is skeletal muscle mass (SMM). Sarcopenia is characterized by low SMM combined with low muscle strength or low physical performance, may be highly prevalent in HNC patients (12). Low SMM seems to have the most significant impact on the incidence of complications, prolonged hospital stay, lower overall survival, lower disease-free survival, and decreased survival in surgical oncology (13).

Weight loss alone cannot predict LBM loss (14), and several studies have underlined the importance of body composition evaluation in HNC patients (8). Besides questionnaires like Patient-Generated Subjective Global Assessment (PG-SGA) that allow the assessment of the nutritional status, weight change, measurement of skinfold thickness, bioelectrical impedance analysis (BIA), computed tomography (CT), magnetic resonance (MRI), or dual-energy x-ray absorptiometry (DXA) are techniques to evaluate nutritional status and/or body composition (10). DXA is considered the gold standard for total body composition analysis (8, 15), but it is a medical exam and not a regular part of the treatment and assessment protocol for HNC patients (10, 12).

Evaluating body composition alterations in HNC patients is essential in order to establish the best therapeutical intervention, but is also a challenging task. We review the existing literature regarding body composition evaluation in HNC patients to determine the most suitable method for this population, regarding availability in the routine clinical practice, patient burden, cost, sensibility, and specificity.

## Methodology

**Figure 1:**
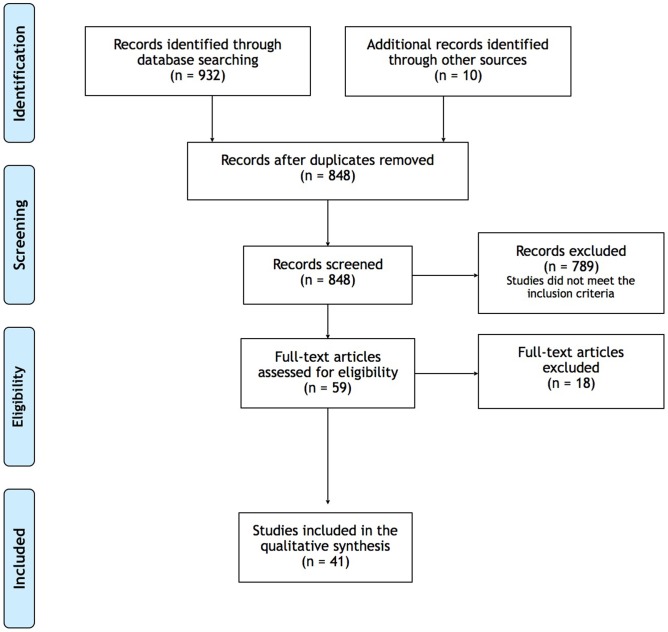
Flow diagram of the study selection process.

A literature search for relevant papers indexed in MEDLINE, Cochrane Library and Scielo was conducted, with no publication date restriction and for all published articles until the 31 January 2019. All the papers wrote in English, with interventions in humans, exclusively considering HNC patients, were selected. Figure 1 describes the selection process of articles. The following conjugated search terms were used: head and neck cancer OR larynx cancer OR hypopharynx cancer OR oropharynx cancer OR lip cancer OR oral cavity cancer or salivary gland cancer OR nasopharynx cancer OR nasal cavity cancer OR paranasal sinus cancer OR middle ear cancer; physical activity; body composition OR bioelectrical impedance analysis OR phase angle OR computed tomography OR magnetic resonance OR dual-energy x-ray absorptiometry OR bioimpedance spectroscopy OR bioelectrical impedance spectroscopy OR BIS OR multiple-frequency BIA OR MFBIA, MF-BIA OR single-frequency OR SFBIA OR BIVA OR bioimpedance vector analysis OR SF-BIA OR bioelectrical impedance vector analysis OR magnetic resonance imaging OR MRI or MRI Scan OR CT scan OR computed tomography scan OR computed axila tomography scan OR DEXA or DXA or CT imaging; LBM OR lean tissue OR FFM OR lean soft tissue OR fat mass OR anthropometric assessment OR body cell mass assessment OR intracellular water.

The first selection of articles was based on the titles and abstracts, and then full texts were evaluated. All the articles that included body composition evaluation using BIA, MRI, CT, DXA, or skinfold measures, in HNC patients over 18 years old, were included in this review. The articles only considering BMI or with body composition evaluations in a population with other cancer sites than the head and neck area, were not included. All study designs were included. Data were collected from each individual study and then systematically recorded in a document that included the following parameters: author, study methodology, study objectives, number of participants, mean age, gender, cancer stage, treatment, median BMI, inclusion and exclusion criteria, body composition assessment (technique, variables assessed, assessment timing), study limitations and conclusions.

## Results

The search of the databases MEDLINE, Cochrane Library and Scielo, resulted in 932 records. After removal of duplicate papers, 848 records were screened, ten papers were found by consulting the references of the screened papers, and after excluding the studies that did not meet the inclusion criteria, 59 full-text articles were assessed for eligibility, 18 of which were excluded for the following reasons:

- Only describing study protocol (*n* = 2);
- Only assessing nutritional status and not body composition (*n* = 4);
- Only assessing phase-angle variations during radiotherapy (RT) (*n* = 1);
- Study population including multiple cancer sites (*n* = 1);
- The final version of the submitted article was not accessible (*n* = 3).

A total of 41 studies were included in this review, with different methodologies: one systematic review (16), one consensus paper (17), one data descriptor (18), nine randomized trials (6, 11, 19–25), 12 cohort studies (3, 7–9, 15, 26–32), three randomized controlled trials (4, 5, 33), three cross-sectional studies (34–36), two prospective non-randomized trials (37, 38), eight retrospective studies (10, 12–14, 39–42), and one case-control study (43).

### Population

The studies included in this review comprised a total of 2,708 participants, 81% were male, and 19% female, with a mean age ranging between 46 and 66 years. Only one study included just male participants (40). The mean baseline BMI ranged from 19.5 to 29.6 kg/m^2^.

The most frequent exclusion criteria were comorbidities (liver, kidney or cardiac disease, chronic obstructive pulmonary disease, muscular disease, or uncontrolled diabetes mellitus), metastatic disease, treatment with steroids and other malignancies.

### Body Composition Assessment

In 15 studies BIA was the assessment method used (3, 5, 7, 8, 20, 21, 23–26, 30, 33, 34, 36, 43), three of which also evaluated skinfolds (23, 25, 30) and one applied bioelectric impedance vector analysis (BIVA) (42). In eight studies, the body composition assessment was made with DXA (4, 6, 9, 11, 19, 22, 29, 31), one of which also included muscle biopsies (9). In two studies, the chosen method was both BIA and DXA (27, 28). CT/ positron-emission tomography-CT (PET-CT) was applied in 11 studies (10, 12–15, 18, 32, 39–42) and one also included MRI (39). In two studies, body composition was assessed with skinfold measures alone (35, 38), one of which also used circumferences (35) and one study only used BIVA (37).

Considering the studies that assessed the patients' body composition with CT/PET, five used the 3rd lumbar vertebra (L3) (10, 14, 18, 32, 42) as a landmark point, one used the 4th lumbar vertebra (L4) (15) and five used the 3rd cervical vertebra (C3) (12, 13, 39–41).

Swartz et al. (12) found a good correlation between the L3 muscle cross-sectional area (CSA) and the C3 muscle CSA in the evaluation of SMM and the method used by these authors was replicated in two other studies (40, 41).

In the studies using C3 as the reference point and calculating the cross-sectional area at L3 (39, 41), the cut-off point for low SMM was <43.2 cm^2^/m^2^, and CSA at L3 was calculated with the formula:

CSA at L3 (cm^2^) = 27.304 + 1.363^*^CSA at C3 (cm^2^)−0.671^*^Age (years) + 0.640^*^Weight (kg) + 26.442^*^Sex (Sex = 1 for female, 2 for male).

One study (40) considered the PVM CSA below 815 mm^2^/m^2^ as a potential prognostic indicator of postoperative wound complications.

Among the studies using BIA as an assessment method, seven did not report the frequencies applied (3, 5, 8, 21, 26, 28, 37); eight used a frequency of 50 kHz (7, 23–25, 30, 33, 34, 43) and three also used other frequencies besides 50 kHz (20, 27, 36). Only two studies mentioned the equations used in the LBM and FBM (Fat Body Mass) calculations (7, 27). None of the studies referred to complications having an association with any of the methods used in the evaluations, such as pain, discomfort, nausea, or dyspnea.

Tables 1–5 resume the information regarding the body composition assessment methods of each study included in this review.

**Table 1:** Body composition assessment: CT scan.

**References**	**Study objective**	**Participants (*n*)**	**Gender (*n*/%)**	**Mean age ± SD**	**Reference point**	**Software**
Nejatinamini et al. (32)	To investigate how vitamin status prior to and after cancer treatment in patients with HNC relates to BC, mucositis, and systemic inflammation	28	M: 23 (82%) F: 5 (18%)	60.3 ± 10.8	L3	Slice-O-Matic software (Slice-O-Matic version 4.3, TomoVision, Magog, QC, Canada)
Grossberg et al. (14)	Characterize the association between skeletal muscle mass depletion and HNSCC survival. Identify and compare the prognostic significance of LBM, weight loss, and BMI on locoregional control and survival	190	M: 160 (84.2%) F: 30 (15.8%)	57.7 ± 9.4	L3	Image-processing platform: Pinnacle 9.6; Philips Medical Systems
Wang et al. (15)	Characterize the changes in body morphomics (total psoas area, lean psoas area, psoas muscle density, HU) before and after chemoradiotherapy by determining the association between these changes with patient-reported quality of life and tumor related outcomes. To determine whether changes in psoas area correlate with changes in total BC as determined by DXA scan	DXA study: 12 BM study: 43 Total: 55	DXA study M: 12 (100%) BM study M: 36 (95%) F: 2 (5%)	DXA study: 57 ± 8.1 BM study: 57 ± 7	L4	MATLAB v13.0
Chamchod et al. (10)	To determine whether formula-based body composition assessment is sufficient as standard practice in the initial work-up and post-therapy surveillance of HNC patients	215	M: 184 (85%) F: 31 (14.4%)	57.21 ± 9.79	L3	Pinnacle 9.6, Philips Medical Systems, Andover, MA
Bril et al. (39)	To investigate whether preoperative low SMM, as measured using CT or MRI at the level of C3, is a significant predictor of postoperative complications	235	M: 193 (82.1%) F: 42 (17.9%)	64.7 ± 9.1	C3	Software package SliceOmatic (Tomovision, Magog, Quebec, Canada)
Bril et al. (13)	To evaluate the interobserver agreement of SMM measurement at the level of C3	54	M: 36 (66.7%) F: 18 (33.3%)	56.8 ± 7.3	C3	Philips Brilliance iCT scanner (Philips Healthcare, Best, The Netherlands)
Swartz et al. (12)	To investigate whether SMM may be assessed on a routine head and neck CT	52	M: 34 (66.7%) F: 17 (33.3%)	61.9 ± 10.5	C3	Volumetool Research software package
Bozkurt et al. (40)	To investigate the relationship between paravertebral muscle cross-sectional area at C3 using CT neck images and complications in advanced laryngeal cancer patients	60	M: 60 (100%)	59.37 ± 8.4	C3	PACS, Infinit Healthcare, South Korea, Guro-gu The CSAs within the limits of the drawn boundaries were calculated using Xelis 3D software (V1.0.6.1, Infinit Healthcare, South Korea, Guro-gu)
Wendrich et al. (41)	To investigate the predictive value of low SMM on chemotherapy dose-limiting toxicity in locally advanced head and neck squamous cell carcinoma patients treated with primary radiochemotherapy. To determine whether low SMM is related to overall survival	112	M: 72 (64.3%) F: 40 (35.7%)	54.5 ± 9.4	C3	Volumetool Research software package
Nishikawa et al. (42)	To investigate the prognostic impact of skeletal muscle depletion and sarcopenia on HNC patients	85	M: 66 (78%) F: 19 (22%)	66	L3	Digital Imaging and Communication in Medicine (DICOM) form ImageJ software v1.44p (National Institutes of Health, Bethesda, MD, USA)
Grossberg et al. (18)	To detail the collection and processing of computed tomography based imaging in 215 patients with HNSCC that were treated with radiotherapy	215	M: 182 (85.5%) F: 33 (15%)	57.2	PET-CT (whole-body) CT (Abdominal - L3)	Pinnacle 9.6; Philips Medical Systems

*BC, body composition; BMI, body mass index; C3, 3rd cervical vertebra; CSA, cross-sectional area; CT, computed tomography; DXA, dual-energy X-ray absorptiometry; F, female; HNC, head and neck cancer; HNSCC, head and neck squamous cell; L3, 3rd lumbar vertebra; L4, 4th lumbar vertebra; LBM, lean body mass; M, Male; MRI, magnetic resonance imaging; PET; positron emission tomography; SMM, skeletal muscle mass*.

**Table 2:** Body composition assessment: DXA.

**References**	**Study objective**	**Participants (*n*)**	**Gender (*n*/%)**	**Mean age ± SD**	**DXA**
Capozzi et al. (19)	To determine the optimal timing for initiation of physical activity intervention	60	M: 49 (81.7%) F: 11 (18.3%)	55.9 ± 9.2	Hologic QDR 4500; Hologic Inc., Bed- ford, MA Hologic QDR software
Jackson et al. (4)	Characterize changes in total BC for patients undergoing concurrent chemoradiation Correlate changes in total BC with hydration status through analysis of serum creatinine levels	12	M: 12 (100%)	57 ± 8.1	iDXA whole body scanner GE/Lunar Corp, Madison, WI
Silver et al. (29)	To investigate changes in BC and energy balance in patients with HNC undergoing concurrent chemoradiation treatment, after completion of low-dose induction chemotherapy	17	M: 15 (88%)	58.9	Lunar Corp Madison, WI Software version 4.3e
Ng et al. (31)	Investigate Nutritional status of nasopharynx cancer patients before and after RT and the factors affecting it	38	M: 30 (78.9%) F: 8 (21.1%)	46	QDA 4500 Elite model, Hologic, Inc., Waltham, MA
Lonkvis et al. (9)	Test the feasibility of a 12-week PRT.To investigate whether PRT may ameliorate weight loss and loss of LBM, maintain muscle strength and functional performance in HNSCC patients	12	M: 7 (58%) F: 5 (42%)	56	GE lunar iDXA, GE Healthcare Technologies, Madison, Wisconsin, US Software version 14.10
Lønbro et al. (6)	To investigate the effects of PRT on LBM in a randomized trial in HNSCC patients following RT	41	Early exercise: F: 5 (31%) M: 11 (69%) Delayed exercise F: 2 (14%) M: 12 (86%)	Early Exercise: 52 ± 7 Delayed Exercise: 58 ± 7	Lunar Prodigy Advance, GE Healthcare Technologies, Madison, WI, USA Scan Analyses: Prodigy enCORE software
Lønbro et al. (11)	To investigate the associations between LBM, maximal muscle strength and functional performance To compare baseline and post-training values of these variables of HNSCC patients to values of healthy individuals	55 HNC (24 healthy individuals)	M: 54 (82%) F: 12 (18%)	56 ± 8	Lunar Prodigy Advance, GE Healthcare Technologies, Madison, WI, USA Scan Analyses: Prodigy enCORE software
Lønbro et al. (22)	To investigate the feasibility of whole body PRT program, protein and creatine supplementation To investigate group changes over time and group differences regarding LBM, muscle strength and functional performance	30	M:23 (76.7%) F: 7 (23.3%)	PROCR group: 56 PLA group: 59	Lunar Prodigy Advance, GE Healthcare Technologies, Madison, WI, USA Software: Prodigy enCORE
Wang et al. (15)	Characterize the changes in body morphomics (total psoas area, lean psoas area, psoas muscle density, HU) before and after chemoradiotherapy by determining the association between these changes with patient-reported quality of life and tumor related outcomes To determine whether changes in psoas area correlate with changes in total BC as determined by DXA scan	DXA study: 12 BM study: 43 Total: 55	DXA study M: 12 (100%) BM study M: 36 (95%) F: 2 (5%)	DXA study: 57 ± 8.1 BM study: 57 ± 7	MATLAB v13.0

*BC, body composition; DXA, dual-energy X-ray absorptiometry; HNC, head and neck cancer; HNSCC, head and neck squamous cell carcinoma; LBM, lean body mass; PRT, progressive resistance training program; RT, radiotherapy*.

**Table 3:** Body composition assessment: review articles.

**References**	**Study objective**	**Articles**	**Body composition (BC) assessment**	**Methods**
Dechaphunkul et al. (17)	Review the literature on HNC to understand how malnutrition and cachexia are defined by researchers publishing in this field	117 articles (14,772 participants)	12/117 articles assessed BC	BIA (*n* = 7), DXA (*n* = 1), anthropometry (*n* = 8)
Capozzi et al. (16)	To systematically summarize the HNC and physical activity literature	16 articles (1,582 participants)	8/16 articles assessed BC	BMI (*n* = 5), DXA (*n* = 4), BIA (*n* = 1), anthropometry (*n* = 1)

*BC, Body composition; BIA, Bioelectrical impedance analysis; BMI, Body mass index; DEXA, Dual-energy X-ray absorptiometry; HNC, Head and neck cancer*.

**Table 4:** Body composition assessment: BIA.

**References**	**Study objective**	**Participants (*n*)**	**Gender (*n*/%)**	**Mean age ± SD**	**BIA model**	**Frequencies (kHz)**	**Equation**	**Description of BIA protocol**
Axelsson et al. (7)	Investigate whether bioelectrical phase angle and standardized phase angle were predictive for survival in advanced HNC	128	M: 87 (68%) F: 41 (32%)	61.4 ± 10.0	Model BIA-101S Akern: RJL Systems, Detroit, MI, USA	50	Lukaski equation	Yes
Lundberg et al. (34)	Describing a cohort of Finnish HNC patients at cancer presentation by medical BIA	41	M: 32 (78%) F: 9 (22%)	62.5	Seca mBCA 515	50	Unknown	No
Solís-Martínez et al. (5)	Assess the effect of the administration of 2 g daily dose of EPA on body composition and inflammation markers in patients with HNSCC during antineoplastic treatment	64	M: 35 (54.6%) F: 29 (46.4%)	58	RJL system using Quantum model IV BC Body Composition Software	Unknown	Unknown	No
Carvalho et al. (30)	Examine the involvement of antitumor treatment, including surgical resection and/or CRT, in the nutritional and metabolic status of patients with HNSCC	32	M: 31 (97%) F: 1 (3%)	NA	BIA 310; Biodynamics, Seattle, WA	Unknown	Unknown	Yes
Della Valle et al. (37)	Evaluate the impact of an early nutritional intervention in patients with HNC with prophylactic gastrostomy undergoing CRT on body weight and body composition	35	M: 20 (57.1%) F: 15 (42.9%)	60	EFG model Akern, Florence, Italy	Unknown	Unknown	No
Weed et al. (20)	Assess the safety and tolerance, as well as the preoperative and postoperative impact of consumption of EPA-containing supplement on weight and BC in adult patients with HNC–related weight loss undergoing treatment with curative intent	31	M: 23 (74.2%) F: 8 (25.8%)	62	BodyStat's Quadscan BodyStat Ltd., Douglas, United Kingdom	5, 50, 150, 200	Standard	Manufacturer instructions
Arribas et al. (8)	To evaluate the changes in BC and nutritional status that occur throughout the oncological treatment in HNSCC patients	20	M: 19 (95%) F: 1 (5%)	53.7 ± 7.11	TANITA BC-418MA segmental Biológica tecología médica, SL, Barcelona, Spain	Unknown	Unknown	Yes
Isenring et al. (21)	Compare the change in BC in ambulatory cancer patients receiving radiotherapy to the head and neck area with groups receiving nutrition intervention or usual care	32	M: 29 (91%) F: 3 (9%)	63 ± 15	BIA foot-to-foot	Unknown	Standard	No
Hopanci Bicakli et al. (3)	To evaluate the effect of compliance with individual dietary counseling provided by the dietitian on BC and anthropometry in HNC patients under RT	59	M: 47 (79.7%) F: 12 (20.3%)	61 ± 13.8	TANITA (Tanita Body Composition Analyzer SC 330 Japan)	Unknown	Unknown	No
Ding et al. (26)	To investigate the longitudinal BC changes in patients with nasopharyngeal carcinoma undergoing CRT. To compare the use of the PG-SGA and the ESPEN diagnostic criteria, in order to explore better BC parameters that could be valuable in diagnosing malnutrition in nasopharyngeal oncology settings	48	M: 36 (75%) F: 12 (25%)	47	nBody S10 Biospace device Biospace Co, Ltd, Seoul, Korea/Model JMW140	Unknown	Unknown	Yes
Grote et al. (33)	To determine the practicability of recruitment and the feasibility of progressive resistance training during RT for cachectic HNC patients	20	M: 15 (75%) F: 5 (25%)	60.9 ± 11.3	AKERN SRL, BIA 101 New Edition	50	Unknown	Yes
Malecka-Massalska et al. (35)	To perform BIA to investigate tissue electrical properties in patients diagnosed with HNC before surgery	31	M: 28 (90.3%) F: 3 (9.7%)	57.9 ± 8.0	iMed bioimpedance analysis SFB7 BioImp, v 1.55 Pinkenba Qld 4008, Australia	5, 50, 100, 200	Unknown	Yes
Luis et al. (24)	To investigate whether postoperative nutrition of HNC patients, using a higher dose of arginine (17 g/day) enhanced diet, could improve nutritional variables as well as clinical outcomes, when compared with a control enteral diet	72	M: 65 (90.3%) F: 7 (9.7%)	Group I: 62.1 ± 12 Group II: 61.5 ± 11	Biodynamics Model 310e, Seattle, WA	50	Unknown	Yes
Luis et al. (43)	To investigate in a case-control study the utility of phase angle and other impedance parameters in a population of male patients with HNC	67	M: 67 (100%)	58.49 ± 14.54	Biodynamics Model 310e, Seattle, WA	50	Unknown	Yes
Luis et al. (25)	To investigate whether oral ambulatory nutrition of HNC patients, using an 3 fatty acid (low ratio 6/3 fatty acids)-enhanced diet versus an oral arginine-enhanced formula, could improve nutritional variables as well as clinical outcome, postoperative infectious and wound complications	73	M: 68 (93.2%) F: 5 (6.8%)	Group I: 60.2 ± 11.15 Group II: 62.5 ± 11.4	Biodynamics Model 310e, Seattle, WA	50	Unknown	Yes
Luis et al. (23)	To investigate whether oral ambulatory nutrition of postsurgical HNC patients with recent weight loss, using two different omega 3 fatty acids enhanced diets could improve nutritional variables as well as clinical outcome	65	M: 59 (90.8%) F: 6 (9.2%)	Group I: 63.9 ± 11.2 Group II: 62.8 ± 11.4	Biodynamics Model 310e, Seattle, WA	50	Unknown	Yes
Jager-Wittenaar et al. (27)	Test the validity of BIA using Geneva equation to assess fat free mass in patients with HNC in pretreatment and post treatment periods	24	M: 20 (83%) F: 4 (17%)	60.4 ± 8.3	Bodystat QuadS-can 4000 (Bodystat)	5, 50, 200	Geneva equation	Yes
Jager-Wittenaar et al. (28)	Test whether nutritional status, including lean body mass, changes during and after HNC treatment including RT or chemoradiation	29	M: 23 (79%) F: 6 (21%)	60.6 ± 10.0	Bodystat QuadS-can 4000 (Bodystat)	Unknown	Unknown	Yes

*BC, body composition, BIA, bioelectrical impedance analysis, CRT, chemoradiotherapy, EPA, eicosapentaenoic acid, ESPEN, European Society for Clinical Nutrition and Metabolism, F, female, HNC, head and neck cancer, HNSCC, head and neck squamous cell cancer, M, male, PG-SGA, Patient-Generated Subjective Global Assessment, RT, radiotherapy*.

**Table 5:** Body composition assessment: Anthropometry.

**References**	**Study objective**	**Participants (*n*)**	**Gender (*n*/%)**	**Mean age ± SD**	**Body composition (BC) assessment**
Corry et al. (38)	Prospective non-randomized trial.	33	M: 24 (73%) F: 9 (27%)	60	Upper arm circumference and triceps skin fold thickness
Fonseca et al. (36)	Cross-sectional	234	M: 211 (90%) F: 23 (10%)	61.6	Upper arm circumference, mid-arm muscle circumference and triceps skin fold thickness

*F, female; M, male*.

## Discussion

Establishing a reliable and easy to use method for body composition assessment in clinical settings remains a challenge. The studies included in this literary review comprised heterogeneous methodologies, objectives as well as methods to assess body composition in HNC patients, which makes it difficult to compare them. We present a review of the existing evidence.

Formula-based body composition assessment (using the Hume formula, Boer formula, and James formula) failed to accurately estimate LBM in HNC patients submitted to radiation treatment when the results were compared with the ones obtained through CT image-based evaluation of L3 (10).

### Anthropometry

Anthropometric measures are widely available, easy to assess and inexpensive. Although it is known that BMI is not sensitive to body composition variations and that in obese populations, it is a poor predictor of muscle mass (10), it is still widely used in clinical settings (44). BMI was also evaluated in all the studies included in this review, and its variations during cancer treatments were reported. With the increase in obesity prevalence in the HNC patient population (10), and as it seems that weight loss alone cannot predict LBM loss (8), methods allowing a more detailed evaluation are needed.

Skinfold thickness measurement allows evaluating subcutaneous fat in sites such as biceps, triceps, subscapular, and supra iliac area using a caliper. One study (38) used it as a single method to assess body composition and aimed to compare 33 patients randomized between a group with a percutaneous endoscopic gastrostomy tube (PEG) and a group with a nasogastric tube for feeding evaluation purposes. Although the authors did not refer to any limitations related to the assessment method, it is known to be sensitive to technician skills, type of caliper and prediction equations used (44). One other study (35) used both skinfold thickness and mid-upper arm (MUAC) and mid-arm muscle circumference (MAMC), to evaluate the outcome and nutritional status at the time of the procedure, of 234 patients who underwent PEG, as well as the association of nutritional status/outcome, creating a predictive survival model. Low MUAC was present in 84% of the patients, and low MAMC was present in 75% of the patients. The authors mention that the slow changes of the anthropometric measurements may make this method inadequate to perceive malnutrition early.

### BIA

Prediction of body composition based on the electrically conductive properties of both lean tissue (good electrical conductor) and fat mass (poor electrical conductor due to the absence of water) is the principle of BIA (44, 45). BIA measures can use single-frequency (SF), which passes through the extracellular fluid or multi-frequency current (MF), which passes through both the extracellular and the intracellular fluid. BIA is an indirect (43) and quick (7) method that estimates total body water (TBW) and through this estimation determines LBM, assuming a constant hydration factor of 73% (33). Fat body mass is calculated from the weight difference between LBM and body weight (33). It is a validated method to assess body composition in patients with cancer (26, 36) and was used in 17 studies in this review.

Both BIA and skinfold measurements were used in three studies (23, 25, 30) that assessed the changes in body composition and nutritional status during cancer treatment with (23, 25) or without (30) nutrition interventions. Foot-to-foot BIA was used by Isenring et al. (21). Despite providing some additional information when compared to BMI or weight loss, it is a method that does not measure the entire body (46) and for that reason gives incomplete information. BIA and BIVA were both used in one study (34), and one other study only used BIVA (37). BIVA is a qualitative evaluation method of hydration, cell integrity and body cell mass that can contribute with additional information to BIA measures. It seems that this (34) was the first study in the population of HNC patients to include BIVA measures. The authors concluded that both BIA and BIVA are useful tools in the assessment of body composition. BIA is an inexpensive method when compared to more sophisticated ones, easy-to-use, non-invasive, and reproducible (26). It has been considered to have good consistency (particularly FFM) in evaluating body composition during HNC treatments (8). However, to enhance accuracy in LBM variations, the evaluations should be done under the same circumstances and taking into consideration an adequate fluid balance (33, 46) and food intake (33). The following possible sources of error should be taken into account: nutrition status, physical activity, phase of the menstrual cycle, placement of electrodes, limb length, blood chemistry (44), altered fluid balance, edema, endocrine diseases that influence body composition, treatment with growth hormone, acute illness, intensive care treatment, organ transplantation, position of the body, and movements during the measure, type of electrodes, use of oral contraceptives (46). This method also loses accuracy when patients are in the extremes of BMI ranges (<= 16 kg/m^2^ or >= 35 kg/m^2^) (7, 46) and although at baseline the patients of the studies included in this review had BMI classifications ranging between normal weight and pre-obesity (with the mean BMI ranging from 21.8 to 29.6 kg/m^2^), it can be a significant limitation in a population susceptible to weight loss during treatments, as HNC patients are (8). Regarding the hydration status, dehydration, or overhydration may underestimate or overestimate LBM or FBM (46), and the studies results are heterogeneous. Luis et al. (43), identified altered electric properties of the tissues in a population of 32 HNC men, but a total body hydration disorder was excluded when comparing the resistance (R) component with one of healthy subjects. On the other hand, Malecka-Massalska et al. (36) mentions a higher electric current resistance due to a smaller distribution of water between the extracellular and intracellular compartments in HNC patients. As an indirect method, it relies on a large number of prediction equations using linear regression to estimate body composition based on a variety of predetermined variables that may differ between different populations and were derived from healthy individuals (46). Two studies (27, 28) in this review used BIA and DXA. One aimed (27) to validate BIA using Geneva equation to assess LBM in 35 HNC patients. In this study, three frequencies were used (5, 50, and 200 kHz) at three-time points (before the start of cancer treatment, 1 and 4 months after the end of treatment). Each BIA measurement was followed by a DXA scan, and the authors only found a slight underestimation (without statistical significance) of LBM using BIA with Geneva equation and considered that this method is acceptable for LBM assessment in this population. The other study was a prospective cohort study with 29 HNC patients. The authors evaluated if the nutritional status changed during HNC treatment. The same previously mentioned three-time points were evaluated, and the alterations in LBM and FBM were registered. No comparison was made between the results obtained with the two methods.

The tendency toward underestimation of both TBW and LBM has already been identified (45, 46). In regards to the oncological patients, Haverkort et al. (46) concluded that BIA estimations could be useful if used longitudinally.

Taking into consideration raw measurements like R, reactance (X) and phase angle can be an advantage in situations in which the equations do not apply as well as to evaluate tissue hydration status (43, 46). This is important as it seems that different BIA devices measuring the entire body, as long as well-calibrated, will give comparable results. However, more studies will be needed to evaluate the clinical applicability of these data (45).

### DXA

DXA measures regional or total body fat, muscle, and bone mineral. It is the gold standard for bone density measurements as well as for determining total body composition (15). It is also a quick method with low radiation exposure and warrants only little preparation and low technical skills (44, 45). DXA is a validated method to assess LBM and body composition in cancer patients (6).

Five studies (4, 6, 11, 19, 22) assessed body composition using DXA to assess the results of a progressive resistance training (PRT) program. The authors reported good feasibility and no complications.

Jackson et al. (4), noted LBM alterations in clinically dehydrated patients and mentioned that the variation in LBM observed on DXA could be a variation in hydration status, as it seems that DXA has failed to determine if changes in hydration occurred in LBM or FBM (4). The variation in hydration status can be a confounding factor, as cancer patients often show fluid status fluctuations, especially during treatments.

Two studies (29, 31) investigated the body composition alterations in HNC patients during cancer treatments and identified the expected decrease in both LBM and FBM.

A single study used both DXA and muscle biopsy (9), in a feasibility test of a 12-week PRT program, including 12 HNC patients. Muscle biopsy was used to analyse further the major alterations registered in LBM during antineoplastic treatments, characterizing muscle fiber types and the enzymes metabolic pathways involved. During treatments, the authors reported a decrease in LBM even during PRT that reverted after the completion of the treatments and mentioned that it was a feasible intervention as a whole and that patients were satisfied with the program.

### CT

CT images are usually part of the routine imaging protocol before and after treatment (4) and CT images of L3 are frequently used in studies assessing body composition in cancer patients (8, 42), as the cross-sectional area (CSA) of L3 have a high correlation to whole body muscle mass (32, 42). It was the chosen method for body composition assessment in four studies (10, 14, 15, 32), which all had different objectives.

The images were mostly taken for diagnostic purposes and after completion of the treatments. The authors of one of the studies identified a considerable weight loss (7,1 kg) with equal losses of muscle (3,4 kg) and fat, during cancer treatment (32) and in another study (14), it was reported that low SMM both before and after treatment is associated with decreased overall and cancer-specific survival.

Body morphomics analysis (BMA) was used in one study (15). It is a CT-based technique that analyses the body composition and characterizes the changes in regional body composition, measuring CSA and densities of the psoas muscle. In this study, the BMA measurements at the level of the L4 were compared to DXA results. The authors found similar results between both techniques and univariate analysis revealed that the total psoas area predicted mortality in oropharyngeal cancer patients.

CT imaging of the L3 is not routinely performed during HNC cancer treatments (8, 13, 14). In fact, some studies pinpoint the exclusion of patients without this exam as a study limitation (15, 42). CT images at the level of C3 may be a cost-effective and a reliable alternative (12, 39, 41), that have the advantage of being a routinely performed imaging method in the majority of HNC patients (39) and in addition causing less radiation exposure and therefore less patient burden. In our review, this method showed a significant correlation with L3 measures considering the sum of paravertebral muscles (PVM) and sternocleidomastoid muscles (SCM).

Although one study (13) included in this review has found an excellent interobserver agreement of SMM measurements at the level of C3 for all CSA measures, the authors advise the use of a training data set to minimize errors in the delineation of SMM and single slice selection. The automatic determination of skeletal muscle area (in a window of −29 to +150 Hounsfield Unit) should be preferred instead of visual delineation of the muscles (12), to avoid overestimation of muscle mass.

Lymph node metastases, which are common in HNC patients at the time of diagnosis, can impair SCM measurement (12, 40). In one study this limitation was minimized, with good results, by doubling the area of the SCM that could be measured (12) and in another study by excluding the SCM from the CSA calculations, as CSA at L3 correlates with CSA PVM alone (40).

In Table 6 are resumed the advantages and limitations of each body composition assessment method in HNC.

**Table 6:** Advantages and limitations of each body composition assessment method in HNC.

**Method**	**Advantages**	**Limitations**
Anthropometry	Widely available Easy to assess Inexpensive	BMI: not sensitive to BC variations; it is a poor predictor of muscle mass in obese populations Skinfold thickness: sensitive to technician skills, type of caliper, and prediction equations used
BIA	Indirect method (relies on prediction equations to estimate BC) Quick Validated to assess BC in patients with cancer Inexpensive Easy-to-use Noninvasive Reproducible	To enhance accuracy in LBM variations, the evaluations should be done under the same circumstances, and taking into consideration an adequate fluid balance and food intake Possible sources of error: nutrition status, physical activity, phase of the menstrual cycle, placement of electrodes, limb length, blood chemistry, hydration status, edema, endocrine diseases that influence body composition, treatment with growth hormone, acute illness, intensive care treatment, organ transplantation, position of the body and movements during the measure, type of electrodes, use of oral contraceptives, extremes of BMI ranges ( ≤ 16 or ≥35 kg/m^2^) Foot-to-foot BIA: does not measure the entire BC
DXA	The gold standard for determining total BC Validated to assess LBM and BC in cancer patients Quick Low radiation exposure Little preparation Low technical skills	Hydration status may influence LBM or fat body mass measures Not routinely performed in the management of HNC
CT	L3: one of the reference methods for BC evaluation in cancer patients C3: routinely performed in the management of HNC	L3: not routinely performed in the management of HNC C3: few studies are available

*BC, body composition; BIA, bioelectrical impedance analysis; BMI, body mass index; C3, 3rd cervical vertebra; CT, computed tomography; DXA, dual-energy X-ray absorptiometry; HNC, head and neck cancer; L3, 3rd lumbar vertebra; LBM, lean body mass*.

## Limitations

This review has some limitations such as the heterogeneity in methodologies, objectives, and assessment methods of the included HNC studies. Only a few studies had as the primary outcome the evaluation of the body composition and not all the studies included detailed results of the performed assessments. Regarding the methods used, there are no validated methods for this specific patient population. Furthermore, the outcome measures can differ between different nationalities.

## Conclusion

This review is a qualitative synthesis of the available evidence regarding body composition assessment methods in HNC patients.

The studies included used different body composition assessment tools, making it challenging to summarize the results.

The reference methods for body composition evaluation in cancer patients are DXA and CT at L3, but these examinations are not routinely performed in the management of HNC. Since variations in body composition in HNC patients are very prevalent, it is of utmost importance to find a tool with low costs and with a low burden to the patient.

Despite the different existing body composition assessment tools, it seems that SMM measurement at the level of C3 may be a reliable method for SMM assessment as it strongly correlates with CSA measures at the level of L3 and it allows a cost-effective body composition assessment without the need for additional radiation exposure.

## Data Availability Statement

The datasets analyzed in this manuscript are not publicly available. Requests to access the datasets should be directed to pedrompneves@gmail.com.

## Author Contributions

IA-C conceived the study, participated in its design and coordination, draft and authored the manuscript. AM and PR participated in the study design, interpretation of the data, and helped to draft manuscript revisions. PN was responsible for scientific writing and manuscript editing. All authors have read and approved the final manuscript.

### Conflict of Interest

The authors declare that the research was conducted in the absence of any commercial or financial relationships that could be construed as a potential conflict of interest.
